# Photoperiodic Regulation of Shoot Apical Growth in Poplar

**DOI:** 10.3389/fpls.2018.01030

**Published:** 2018-07-13

**Authors:** Paolo M. Triozzi, José M. Ramos-Sánchez, Tamara Hernández-Verdeja, Alicia Moreno-Cortés, Isabel Allona, Mariano Perales

**Affiliations:** ^1^Centro de Biotecnología y Genómica de Plantas, Universidad Politécnica de Madrid-Instituto Nacional de Investigación y Tecnología Agraria y Alimentaria Madrid, Spain; ^2^Departamento de Biotecnología-Biología Vegetal, Escuela Técnica Superior de Ingeniería Agronómica, Alimentaria y de Biosistemas, Universidad Politécnica de Madrid Madrid, Spain

**Keywords:** poplar, photoperiodic time measurement, seasonal growth, shoot apical growth, circadian clock, flowering locus T, tempranillo, constans

## Abstract

Woody perennials adapt their genetic traits to local climate conditions. Day length plays an essential role in the seasonal growth of poplar trees. When photoperiod falls below a given critical day length, poplars undergo growth cessation and bud set. A leaf-localized mechanism of photoperiod measurement triggers the transcriptional modulation of a long distance signaling molecule, FLOWERING LOCUS T (FT). This molecule targets meristem function giving rise to these seasonal responses. Studies over the past decade have identified conserved orthologous genes involved in photoperiodic flowering in Arabidopsis that regulate poplar vegetative growth. However, phenological and molecular examination of key photoperiod signaling molecules reveals functional differences between these two plant model systems suggesting alternative components and/or regulatory mechanisms operating during poplar vegetative growth. Here, we review current knowledge and provide new data regarding the molecular components of the photoperiod measuring mechanism that regulates annual growth in poplar focusing on main achievements and new perspectives.

## Introduction

Shoot apical growth in poplar is extremely sensitive to day length (Howe et al., 1995). Under long day conditions, internode elongation and shoot organogenesis occur continuously. However, when photoperiod falls below a critical day length (CDL), these events come to a halt and this phenomenon is known as growth cessation (Weiser, 1970; Thomas and Vince-Prue, 1997). Poplar annual growth is therefore controlled by a photoperiodic time measurement (PTM) mechanism. This molecular mechanism is able to recognize seasonal photoperiod information by monitoring regular changes in day or night duration. Thus, the components of PTM must be under the control of light signaling pathways. A pulse of red illumination during the night abolishes short day-induced poplar growth cessation (Howe et al., 1996). Similar night break experiments have been found to accelerate Arabidopsis flowering under conditions of short days (Reed et al., 1994). Such responses to night break experiments suggest that poplar shoot growth and Arabidopsis flowering share a similar photoperiod regulation mechanism. In effect, functional studies have suggested that the genetic control of Arabidopsis flowering time and poplar shoot apical growth is conserved (Böhlenius et al., 2006; Hsu et al., 2011; Ding et al., 2018). However, in the present study we show that some features of the PTM mechanism such as the daily expression pattern and molecular function of the *FT* repressor *TEMPRANILLO* (*TEM*) vary between the two plant models. We also describe that by comparing diurnal gene expression with height-associated single nucleotide polymorphism (SNP) genes, we here identified *GIGANTEA* (*GI*) and *FLAVIN BINDING, KELCH REPEAT, F-BOX1* (*FKF1*), already shown to be involved in poplar seasonal growth (Ding et al., 2018), along with new candidate orthologous genes to Arabidopsis flowering time regulators. Interestingly, the genes *VERNALIZATION INDEPENDENCE 4* and *5* (*VIP4* and *VIP5*), *AGAMOUS-LIKE* (*AG-like*) and *TERMINAL FLOWER 2* (*TFL2*) associated with the vernalization pathway in Arabidopsis, show robust diurnal rhythms of mRNA accumulation in poplar, suggesting their potential role as photoperiodic regulators of poplar shoot apical growth.

## A PTM mechanism controls seasonal development

Photoperiod is the most regular environmental signal that drives seasonal development in many insects, birds, other animals and plants (reviewed in Nelson et al., 2010). The external coincidence hypothesis, initially proposed by Bünning (1936) and later modified by Pittendrigh and Minis (1964), has been the prevailing PTM model tested in many organisms (Goldman, 2001; Pegoraro et al., 2014; Song et al., 2015). This model predicts that seasonal physiological responses are created when, by coincidence, diurnal endogenous rhythms match the external photoperiod. Accordingly, day/night duration is measured through photoperiod-dependent modulation of the activity of an endogenous oscillatory component, which controls seasonal physiologic and developmental responses (Pittendrigh and Minis, 1964). Three basic components are required to create a day length measurement mechanism: (1) a photosensory system which includes photoreceptors, (2) an endogenous oscillator, which has been identified as the circadian clock system, and (3) an endocrine effector or mobile photoperiodic signal that translates photoperiod information from the photosensory system to the target organs. In plants, the molecular nature of these components was firstly identified for Arabidopsis flowering time (Yanovsky and Kay, 2002). In woody perennials, components of the PTM mechanism have been recently identified in poplar (reviewed in Maurya and Bhalerao, 2017). In the sections below, we review the state of the art of this research topic.

## Phytochromes as players in the poplar PTM mechanism

It is assumed that the photoperiodic signal is perceived in the leaf through discrimination of light quality information by photoreceptors, which have been linked to growth regulation in poplar (Howe et al., 1996; Olsen et al., 1997; Frewen et al., 2000; Ingvarsson et al., 2008; Ruonala et al., 2008; Kozarewa et al., 2010). In carefully-designed night-break experiments, subjecting plants to red light illumination caused the suppression of short day-induced growth cessation in poplar. This effect was reversed by red light followed by far red light night-break treatment, indicating that phytochromes participate in the photoperiodic regulation of poplar shoot apical growth (Howe et al., 1996). One phytochrome A (*PHYA*) and two phytochrome B (*PHYB*) genes, *PHYB1* and *PHYB2*, were identified in poplar (Howe et al., 1998). The overexpression of oat *PHYA* in hybrid aspen (*Populus tremula x tremuloides*) showed relative insensitivity to photoperiod-induced growth cessation (Olsen et al., 1997). Conversely, hybrid aspen showing downregulation of *PHYA* has been noted to cease growth earlier than control plants subjected to photoperiod-inducing conditions below CDL (Kozarewa et al., 2010). Poplar *PHYB1* but not *PHYB2* has been observed to complement an Arabidopsis *phyB* mutant indicating that PHYB1 maintains the molecular function of Arabidopsis PHYB, while PHYB2 shows divergent molecular features (Karve et al., 2012). However, quantitative trait loci and single nucleotide polymorphism analyses have genetically linked the timing of photoperiod-induced bud set to *PHYB2*, but not to *PHYB1* (Frewen et al., 2000; Ingvarsson et al., 2008). Thus, PHYA and PHYB2 could play a photosensory function during poplar shoot apical growth and may participate in the PTM mechanism.

Some authors have explored the link between PHYA and the circadian clock. Interestingly, poplar *PHYA* antisense gave rise to a longer period of leaf movement rhythms than wild type plants indicating that the circadian clock period depends on the PHYA level (Kozarewa et al., 2010).

## Poplar circadian clock genes participate in photoperiodic regulation of shoot apical growth

The circadian clock is an endogenous molecular oscillator that creates a 24 h rhythmic pattern of gene expression, physiology, cell division and development (reviewed in Nohales and Kay, 2016). The first clock genes identified in a woody perennial species were chestnut *LATE ELONGATED HYPOCOTYL (LHY)* and *TIMING OF CAB EXPRESSION 1 (TOC1)*, orthologs of essential components of the Arabidopsis circadian clock (Ramos et al., 2005). Later, the homologous Arabidopsis genes *PSEUDO–RESPONSE REGULATORS (PRR), PRR9, PRR7, and PRR5* were identified in chestnut and these genes showed daily peak expression after *LHY* in the order *PRR9*→*PRR7*→*PRR5*→*TOC1*, in a similar serial manner to that seen in Arabidopsis (Ibáñez et al., 2008).

Poplar genes of the circadian clock system have been identified based on sequence homology with their Arabidopsis orthologs (reviewed in Johansson et al., 2015). Poplar has two copies of the *LHY* transcription factor, denoted *LHY1* and *LHY2* (Takata et al., 2009). The daily gene expression pattern of poplar *LHY1* and *LHY2* shows a morning expression peak similar to Arabidopsis and chestnut (Takata et al., 2009; Ibáñez et al., 2010; Ramos-Sánchez et al., 2017). Moreover, the poplar clock has several *PRR* orthologs of Arabidopsis *PRR5, PRR7, PRR9* and *PRR1/TOC1*, which display a similar daily gene expression pattern to that previously reported in Arabidopsis and chestnut (Ramos et al., 2005; Ibáñez et al., 2008, 2010; Filichkin et al., 2011). Recently, two poplar orthologs of the Arabidopsis clock gene *GI*, designated *GI* and *GIL*, and two F-box protein orthologs of the clock regulator *FKF1*, denoted *FKF1a* and *FKF1b*, have been identified (Ding et al., 2018).

Pioneer functional analyses of poplar *LHY* and *TOC1* clock genes revealed that the downregulation of *LHY* or *TOC1* caused a drastic delay in growth cessation with respect to the wild type, confirming that circadian clock function is needed for seasonal regulation of growth (Ibáñez et al., 2010). Remarkably, both *LHY* and *TOC1* RNA interference (RNAi) plants showed a reduced period relative to their wild type counterparts, the period in *LHY* RNAi being much shorter than in *TOC1* RNAi (Ibáñez et al., 2010). This observation prompts the interesting question of how the PTM mechanism works. Thus, it could be shortening of the internal period or reduced levels of *LHY* or *TOC1* expression or both together that were responsible for the observed late growth cessation phenotype in *LHY* and *TOC1* RNAi plants.

## FT2 acts as mediator of photoperiodic signaling to control poplar shoot apical growth

**Figure 1:**
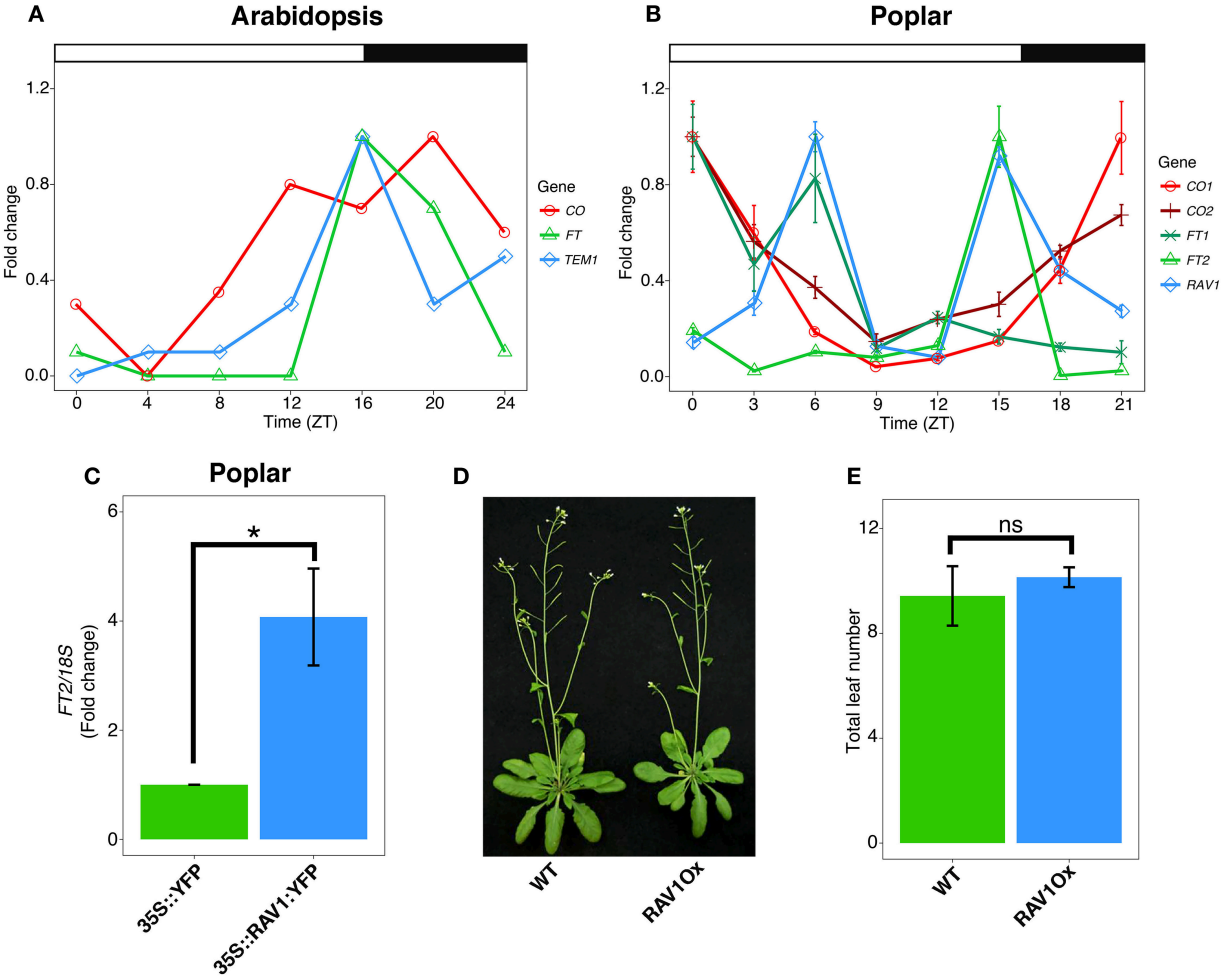
Divergent temporal expression pattern and molecular function of Poplar *TEM/RAV1*. **(A)** Representative diurnal mRNA expression pattern of Arabidopsis *FT, CO* and *TEM* under long day conditions. **(B)** Diurnal mRNA expression of *FT1, FT2, CO1, CO2*, and *RAV1* examined through qRT-PCR of hybrid poplar (*Populus tremula x alba*) wild-type leaves under long day conditions. **(A,B)** Gene expression was relativized to the maximum value for each gene and represented as fold change to compare the diurnal patterns. Noteworthy, maximum expression peaks of *FT2* and *CO1* are 24 and 3.5 times higher than *FT1* and *CO2*, respectively. Time is expressed in hours from dawn (ZT, zeitgeber time). Error bars indicate the standard deviation corresponding to three technical replicates. **(C)** *FT2* mRNA expression at ZT15. qRT-PCR analysis performed on hybrid poplar leaf samples transiently expressing *35S::RAV1:YFP::tNOS* and *35S::YFP::tNOS* (control) constructs. Data are represented as the mean ± se of three independent experiments. Asterisk indicates significant differences between the *RAV1* overexpressing (Ox) construct and control (*t*-test, *P* < 0.05). **(D)** Representative picture showing the flowering of wild type and chestnut RAV1Ox Arabidopsis plants grown for 5 weeks under long day conditions. **(E)** Flowering time of wild type and chestnut RAV1Ox Arabidopsis plants obtained by counting the total leaf number at bolting transition of plants grown under long day conditions (*n* = 12). Error bars indicate the standard deviation of the mean. n.s., not significant (*t*-test, *P* > 0.05).

The mobile protein FT transmits photoperiod information from the leaf to apex to control Arabidopsis flowering (Corbesier et al., 2007). *FT* gene transcription is extremely sensitive to photoperiod changes. Long days enhance *FT* transcription and short days drastically reduce it. *FT* shows a robust diurnal gene expression pattern under long day conditions showing a peak of mRNA accumulation at the end of the day (Figure 1A). This temporal pattern is critical to distinguish photoperiod information from other signals (Krzymuski et al., 2015). The combined actions of circadian clock-controlled activators and repressors sustain this tight *FT* diurnal expression in Arabidopsis (reviewed in Song et al., 2015).

Two poplar orthologs of Arabidopsis *FT* have been identified. These show different spatio-temporal pattern of gene expression whereby *FT1* is mainly expressed in stem and bud tissues at the end of winter, and *FT2* expression occurs during the growing season mainly in leaf tissues (Pin and Nilsson, 2012). Remarkably, poplar *FT2* mRNA levels indicate conserved diurnal expression and photoperiod responses as in Arabidopsis (Böhlenius et al., 2006; Ibáñez et al., 2010; Hsu et al., 2011; Figure 1B). Moreover, natural variation across a latitudinal gradient suggests diurnal *FT2* expression is locally adapted to the day length regime (Böhlenius et al., 2006). Further, the ectopic expression of *FT2* in poplar has been noted to induce a dramatic delay in growth cessation. Conversely, *FT2* downregulation significantly speeds up growth cessation (Böhlenius et al., 2006; Hsu et al., 2011). These studies have shown the conserved role of poplar FT2 as a mediator of the photoperiod signaling required for shoot apical growth. Whether mobile FT2 transfers photoperiod information to the poplar shoot apical meristem (SAM) is unknown. However, recent genetic studies indicate that trafficking of FT, or an FT-dependent signal, to the SAM is mediated by plasmodesmata (Tylewicz et al., 2018). Downregulation of *FT2* is necessary but not sufficient for poplar growth cessation. Resman et al. (2010) showed that the timing of growth cessation involved additional downstream events to FT2. However, whether these operate under the control of an FT-dependent PTM mechanism remains to be investigated.

## Diurnal patterns of poplar *CONSTANS* and *RAV1/TEMPRANILLO* suggest an alternative mechanism of transcriptional *FT2* regulation

The balance between the transcriptional activation role of *CONSTANS* (*CO*) and the repressive activity of *TEM* is critical to maintain adequate *FT* mRNA levels (Suárez-López et al., 2001; Castillejo and Pelaz, 2008). CO expression is controlled by the circadian clock, and diurnal transcriptional activation of *CO* precedes the activation of *FT* under long day conditions (Figure 1A). Mutations in *lhy-7* and *lhy-20*, which shorten the clock period, have been detected to induce the early timing of *CO* expression and high levels of *FT* (Park et al., 2016). Hence, the diurnal matching of *CO* and *FT* expression patterns is important to control Arabidopsis flowering. CO protein levels are also diurnally regulated. Accordingly, during daylight hours, CO protein levels are maintained, while in the dark, CO levels are reduced contributing to *FT* downregulation (Valverde et al., 2004). Moreover, the repression of *FT* is transcriptionally controlled by TEM, a transcription factor related to APETALA 2 and VIVIPAROUS-1 (RAV1), which directly binds the *FT* promoter (Castillejo and Pelaz, 2008). *TEM* and *FT* are co-expressed, showing peak mRNA accumulation at the day-night transition when days are long (Castillejo and Pelaz, 2008; Osnato et al., 2012; Figure 1A).

Poplar orthologs of *CO* have been identified and functionally implicated in the photoperiodic regulation of poplar shoot growth and flowering (Böhlenius et al., 2006). Simultaneous downregulation of poplar *CO1* and *CO2* correlates with reduced levels of poplar *FT2* leading to accelerated growth cessation under short day conditions (Böhlenius et al., 2006). In Arabidopsis, the ectopic expression of *CO* gives rise to constitutively high levels of *FT* leading to early flowering under conditions of both long and short days. Consequently, in these plants, high-levels of *CO* expression are sufficient to promote flowering (Putterill et al., 1995). In contrast, the overexpression of poplar *CO1* or *CO2* orthologs was found neither to upregulate *FT2* nor delay growth cessation under conditions of short days in poplar (Hsu et al., 2012), suggesting that *FT2* repression is dominant under short day conditions. Moreover, diurnal expression patterns of poplar *CO1* and *CO2* show an anti-phase temporal pattern relative to *FT2*, indicating an alternative mode of action to the Arabidopsis model (Figure 1B). Recently, Ding et al. (2018) showed that *GI* contributes to poplar photoperiodic control of shoot apical growth via strong direct *FT2* activation, whereas in Arabidopsis, GI strongly activates both *CO* and *FT* (Sawa and Kay, 2011; Ding et al., 2018). Poplars overexpressing GI show delayed short day-induced growth cessation indicating its predominant role as an activator of *FT* over CO (Ding et al., 2018).

Interestingly, a poplar *RAV1* ortholog of the *TEM* gene has been also identified (Moreno-Cortés et al., 2012). QRT-PCR expression analysis of poplar *RAV1* has revealed a different diurnal pattern from that observed for Arabidopsis *TEM* (Figures 1A,B). Thus, in leaves, poplar *RAV1* shows two mRNA peaks, the first in the morning and the second in the evening, the later overlapping with the *FT2* peak (Figures 1A,B). Unexpectedly, in a transactivation assay in poplar leaf tissues, the overexpression of *RAV1* led to *FT2* activation (Figure 1C). Supporting this observation, the overexpression of the chestnut *RAV1* ortholog of *TEM* did not cause delayed flowering in Arabidopsis (Moreno-Cortés et al., 2012, Figures 1D,E). These data suggest that hybrid poplar and chestnut *TEM* orthologs do not operate as in Arabidopsis.

While the expression pattern and function as an integrator of photoperiodic information of *FT2* is well-conserved in Arabidopsis and poplar (Böhlenius et al., 2006; Hsu et al., 2006, 2011; Tylewicz et al., 2015, 2018), some known *FT2* regulators show different modes of action. This suggests that the molecular framework for poplar seasonal growth could involve additional players with particular and diverged features.

Photoperiod signaling downstream from FT2, controls the rate of shoot apical cell proliferation (Karlberg et al., 2010). FT2 targets a poplar ortholog of Arabidopsis APETALA1, *Like-AP1* (*LAP1*), via interaction with the FLOWERING LOCUS D poplar ortholog (FDL1) (Tylewicz et al., 2015). LAP1 activity maintains shoot vegetative growth via direct regulation of *AINTEGUMENTA LIKE 1* (*AIL1*) transcription factor (Azeez et al., 2014). Interestingly, the ectopic expression of LAP1 in poplar does not promote early flowering as shown for Arabidopsis AP1, pointing to diverged features for LAP1 in poplar (Mandel et al., 1992; Azeez et al., 2014).

## Diurnal gene expression of height-associated SNPs uncovers potential photoperiodic regulators of poplar shoot apical growth

**Figure 2:**
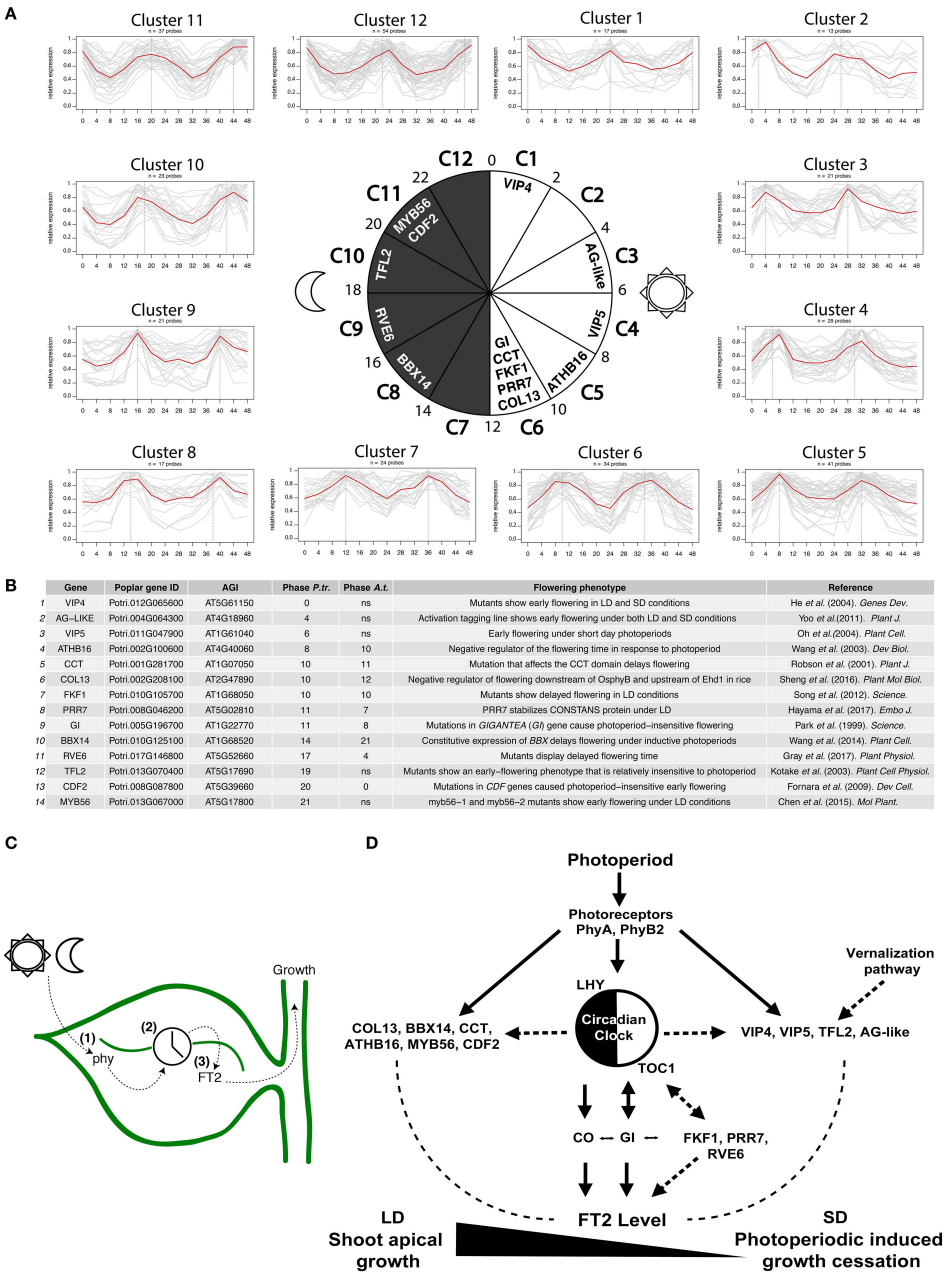
Diurnal oscillations of poplar genes showing height-associated SNPs serve to unravel potential regulators of the photoperiodic control of shoot apical growth. **(A)** Clusters of poplar genes showing height-associated SNPs based on the phase of peak expression from the diurnal expression database. A total of 12 clusters were obtained covering the 24 h of the day and grouping phases every 2 h starting at cluster 1 (including phases 0 and 1). The central circadian circle shows the temporal succession of the clusters including identified poplar orthologs of known Arabidopsis flowering time regulators. **(B)** List of poplar genes orthologous to Arabidopsis flowering time regulators. Arabidopsis mutations in these genes gave rise to altered expression levels of *FT*. Poplar and Arabidopsis expression phases are shown (12:12 h light/dark, LDHC, cutoff 0.8). **(C)** Schematic representation of the leaf-localized photoperiod measurement mechanism in poplar. (1) Photosensory pathway, (2) circadian clock system, and (3) mobile photoperiodic mediator signal. **(D)** Schematic representation of poplar known and predicted photoperiod measurement components. Continuous lines represent experimentally supported links in poplar. Dashed lines represent experimentally supported links in Arabidopsis.

To examine whether the diurnal regulation of poplar shoot apical growth involves additional conserved known Arabidopsis flowering time regulators, we explored the diurnal expression of genes showing height-associated SNPs (Mockler et al., 2007; Evans et al., 2014). A total of 17% of genes associated with height showed robust diurnal rhythms of transcription over a 48 h period, indicating the requirement of diurnal gene expression for poplar shoot apical growth. We detected clusters of genes showing all possible diurnal expression patterns, indicating no specific phase enrichment in diurnally-controlled height increase (Figure 2A). Individual clusters were examined for poplar orthologs of flowering time genes that have been attributed a regulating role in the transition from vegetative growth to flowering in Arabidopsis. This led to the identification of 14 genes, whose altered expression in Arabidopsis gives rise to a variation in flowering time (Figures 2A,B). Interestingly, orthologs of the Arabidopsis clock-controlled *GI, PRR7, FKF1*, and *REVEILLE 6* (*RVE6*) confirmed the role of the circadian clock in the regulation of shoot apical growth. Similarities in timing of mRNA accumulation peaks shown by Arabidopsis *GI, FKF1*, and *PRR7* and the poplar orthologs suggest conserved transcriptional regulation despite the speciation process (Park et al., 1999; Song et al., 2012; Gray et al., 2017; Hayama et al., 2017) (Figures 2A,B). Phase differences in daily rhythms reflect local environmental constraints, which prompt diversification of molecular function contributing to phenotypic variation in natural populations (de Montaigu and Coupland, 2017). The poplar *GI* phase is delayed only 3 h compared with Arabidopsis *GI* and is synchronous with the daily timing of poplar *FT2* activation (Figures 2A,B). The roles of *FKF1, PRR7*, and *RVE6* in the photoperiodic regulation of poplar shoot apical growth requires further investigation. Physical interaction between FKF1 and GI has been reported in poplar, while the implications of this interaction remain unclear (Ding et al., 2018).

Additionally, we have identified 3 poplar CCT domain (CO, CO-like, and TOC1)-containing transcription factors designated *COL13, BBX14*, and *CCT*, showing both height-associated SNPs and robust diurnal expression (Robson et al., 2001; Wang et al., 2014; Sheng et al., 2016) (Figures 2A,B). Proteins carrying the CCT domain have been implicated in the photoperiodic regulation of *FT* in Arabidopsis. Moreover, the CCT motif has been shown to bind DNA and participate in protein-protein interactions (Wenkel et al., 2006; Gendron et al., 2012). Poplar *COL13* and *CCT* show a phase similar to *GI* (Figures 2A,B). However, it remains to be determined whether they are also involved in the activation of *FT2* in poplar.

A poplar ortholog of Arabidopsis *CYCLING DOF FACTOR 2* (*CDF2*) was also found within cluster 11 (Figure 2A). This factor has been shown to transcriptionally repress *CO* (Fornara et al., 2009; Figure 2B). The overexpression of poplar *CDF3* causes repression of *FT2* and earlier growth cessation (Ding et al., 2018). Further, CDF2 was found to physically interact with GI and FKF1 in yeast two-hybrid assays, though in future work it remains to be elucidated if this interaction is meaningful (Ding et al., 2018).

A poplar ortholog of *ARABIDOPSIS HOMEBOX 16* (*ATHB16*), which is a member of the HD-ZIP family of plant transcription factors, shows a similar expression phase in both plant species (Figures 2A,B). Functional studies have shown that ATHB16 acts as a negative regulator of flowering time in Arabidopsis (Figure 2B). Genetics studies have located ATHB16 downstream of the blue light signaling pathway (Wang et al., 2003). However, it is still not known if this pathway contributes to the control of poplar shoot apical growth.

We also identified a poplar ortholog of the Arabidopsis transcription factor *MYB56*, which has been shown to be a negative regulator of photoperiodic flowering time and *FT* expression in Arabidopsis (Chen et al., 2015). Interestingly, this poplar ortholog shares identical diurnal gene expression with the poplar ortholog of CDF2 and could thus be a negative regulator of poplar *FT2* and growth cessation (Figures 2A,B). Contrary to findings in poplar, the diurnal expression of *MYB56* showed no significant rhythmicity in Arabidopsis suggesting its different regulation in trees.

Remarkably, poplar orthologs of Arabidopsis *VIP4* and *VIP5, AG-like* and *TFL2*, all associated with plant height, have also shown robust diurnal rhythms in poplar. However, diurnal variations in Arabidopsis *VIP4, VIP5, AG-like*, and *TFL2* were found not to be significant, indicating their orthologous poplar genes have acquired robust diurnal expression (Figures 2A,B). Arabidopsis VIP4, VIP5, AG-like, and TFL2 have been attributed roles in flowering time and vernalization through the epigenetic regulation of *FLOWERING LOCUS C* (*FLC*) and *FT*, particularly for TFL2 (Kotake et al., 2003; He et al., 2004; Oh et al., 2004; Yoo et al., 2011). It would be interesting to determine whether poplar orthologs of *VIP4, VIP5, AG-like*, and *TFL2* participate in the regulation of shoot apical growth via rhythmic deposition and recognition of epigenetic marks in poplar.

Collectively, the available data have served to identify several poplar orthologs of Arabidopsis photoperiod-controlled flowering time regulators that could play a role in poplar shoot apical growth. Interestingly, we found key regulators of the vernalization pathway that show robust diurnal rhythms in poplar. This reveals that the transcriptional regulation of *FT* in Arabidopsis and poplar could share a larger molecular framework than was previously envisaged and new regulatory pathways (Figures 2C,D).

## Concluding remarks

Poplar has a PTM mechanism that controls seasonal growth. This mechanism preserves the basic functions and molecular components of the mechanism of flowering time regulation known for Arabidopsis. Elements of the photosensory pathway, the circadian clock, and a mobile photoperiodic mediator are prerequisites for an adequate poplar growth cessation response (Figure 2C). Diurnal expression patterns serve to track the photoperiodic signal. Diurnally- expressed genes featuring SNPs associated with height show all possible diurnal phases. Divergent expression patterns of known and predicted PTM components relative to those of Arabidopsis suggest different modes of action in poplar. Interactions among photoreceptors, circadian clock system and *FT2* regulation in poplar need further investigation. We here propose new potential candidates (Figure 2D). Among these candidates, we would highlight the poplar orthologs of epigenetic regulators of the Arabidopsis vernalization pathway. These factors show an unusually robust diurnal expression pattern in poplar, suggesting they could play a critical role in the photoperiodic pathway.

## Author contributions

PT, IA, and MP planned and designed the research. PT, TH-V, and AM-C performed experiments and analyzed data. PT, JR-S, IA, and MP wrote the manuscript. All authors read and approved the final manuscript.

### Conflict of interest statement

The authors declare that the research was conducted in the absence of any commercial or financial relationships that could be construed as a potential conflict of interest.
